# Interacting effects of obesity, race, ethnicity and sex on the incidence and control of adult-onset asthma

**DOI:** 10.1186/s13223-016-0155-8

**Published:** 2016-10-19

**Authors:** Corinna Koebnick, Heidi Fischer, Matthew F. Daley, Assiamira Ferrara, Michael A. Horberg, Beth Waitzfelder, Deborah Rohm Young, Michael K. Gould

**Affiliations:** 1 Department of Research and Evaluation, Kaiser Permanente Southern California, 100 S. Los Robles, 2nd Floor, Pasadena, CA 91101 USA; 2 Institute for Health Research, Kaiser Permanente Colorado, 10065 E. Harvard Street Suite 300, Denver, CO 80231 USA; 3 Division of Research, Kaiser Permanente Northern California, 2000 Broadway, Oakland, CA 94612 USA; 4 Mid-Atlantic Permanente Research Institute, Kaiser Permanente Mid-Atlantic States, 2101 East Jefferson Street 3 West, Rockville, MD 20852 USA; 5 Center for Health Research-Hawaii, Kaiser Permanente Hawaii, 501 Alakawa Street Suite 201, Honolulu, HI 96817 USA

**Keywords:** Asthma, Adult-onset, Obesity, Race, Sex, Ethnicity

## Abstract

**Background:**

To improve care and control for patients with adult-onset asthma, a better understanding of determinants of their risk and outcomes is important. We investigated how associations between asthma, asthma control and obesity may be modified by patient demographic characteristics.

**Methods:**

This retrospective study of adults enrolled in several health plans across the U.S. (n = 2,860,305) examined the interacting effects of obesity, age, race, and sex on adult-onset asthma and asthma control. Multivariable adjusted Cox and logistic regression models estimated hazard ratios (HR), and 95 % confidence intervals (CI) for the associations between body mass index (BMI) and study outcomes, and interactions of BMI with demographic characteristics.

**Results:**

Compared with individuals who had a BMI <25 kg/m^2^, the hazard of adult-onset asthma progressively increased with increasing BMI, from a 12 % increase among persons with a BMI of 25.0–29.9 kg/m^2^ (HR 1.12, 95 % CI 1.10, 1.14) to an almost 250 % increase among persons with a BMI ≥50 kg/m^2^ (HR 2.49, 95 % CI 2.38, 2.60). The magnitude of the association between obesity and asthma risk was greater for women (compared with men) and lower for Blacks (compared with non-Hispanic Whites). Among individuals with asthma, obesity was associated with poorly controlled and high-risk asthma.

**Conclusions:**

The present study demonstrates that the magnitude of the associations between obesity and adult-onset asthma incidence and control are modified by race, age, and sex. Understanding the role of obesity in the development of adult-onset asthma will help to improve asthma treatment algorithms and to develop targeted interventions.

## Background

Asthma is a chronic inflammatory disorder of the airways that involves a complex interaction of airflow obstruction, bronchial hyperresponsiveness, and underlying inflammation [1]. The prevalence of asthma in the United States is about 9 % in women and 7 % in men [2]. While asthma often starts in early childhood, adult-onset asthma is often associated with poor control, more symptoms, and higher medication needs than pediatric-onset asthma [3]. To improve care and control for patients with adult-onset asthma, a better understanding of determinants of their risk and outcomes is important.

While several previous studies have shown that obesity is a risk factor for asthma [4], recent studies also indicate that patients with asthma who are obese may represent a clinically distinct subset of patients [5]. Compared to patients with asthma who are normal or overweight, patients with asthma who are obese have more severe and frequent respiratory symptoms, greater exacerbation rates and reduced asthma-related quality of life [6]. Because most studies have not distinguished between pediatric and adult-onset asthma, little is known about the risk and outcomes of adult-onset asthma, the role of obesity in asthma development, and how the risk of asthma among the individuals who are obese may be modified by other factors such as race/ethnicity and sex. Understanding the role of obesity in developing adult-onset asthma and achieving control across heterogeneous groups of patients will help to improve asthma control algorithms and to develop targeted interventions. This knowledge will foster future patient-centered outcome studies to better understand patient barriers to asthma control among patients with asthma who are affected by obesity.

In this large, population-based cohort study using electronic health record (EHR) data of 2.8 million individuals who were members of nine different health plans across the United States, we examined the association between body weight and the incidence of adult-onset asthma. Among adults with incident adult-onset asthma, secondary outcomes such as poorly controlled asthma with or without high asthma medication ratio (AMR) as indicator for difficulties to achieve asthma control, and high-risk asthma, were examined. In addition, we examined race and sex as potential modifiers of the effect of body weight on asthma risk and outcomes.

## Patients and methods

### Study setting

This study was conducted by the patient outcomes research to advance learning (PORTAL) network. The health care systems in the PORTAL network collectively include 11 million members, as previously described [7, 8]. In brief, PORTAL includes Kaiser Permanente, Group Health Cooperative, HealthPartners, and Denver Health. The cohort is racially and socioeconomically diverse, and generally representative of the underlying populations of the health plans’ service regions [9, 10]. All PORTAL sites contributed data for the present study except HealthPartners.

The study protocol was reviewed and approved by the Institutional Review Board (IRB) of Kaiser Permanente Southern California (KPSC) as the lead site. The IRBs at the other sites reviewed the protocol and subsequently ceded review to the KPSC IRB.

### Study design and population

We performed a retrospective cohort study using the PORTAL overweight/obesity cohort, which includes over 5 million adults with a BMI ≥23.0 kg/m^2^ in 2012–2013. A detailed description of the cohort is provided elsewhere [8]. Briefly, the PORTAL overweight/obesity cohort consisted of individuals who were 18 years of age and older when entering the cohort, had 1 year of continuous membership in any of the contributing health plans prior to entering the cohort and had at least one outpatient medically-related visit with a biologically plausible recorded measure of weight and height with a BMI of ≥23.0 kg/m^2^ at any time between 2012 and 2013 (n = 5,293,458).

**Fig. 1:**
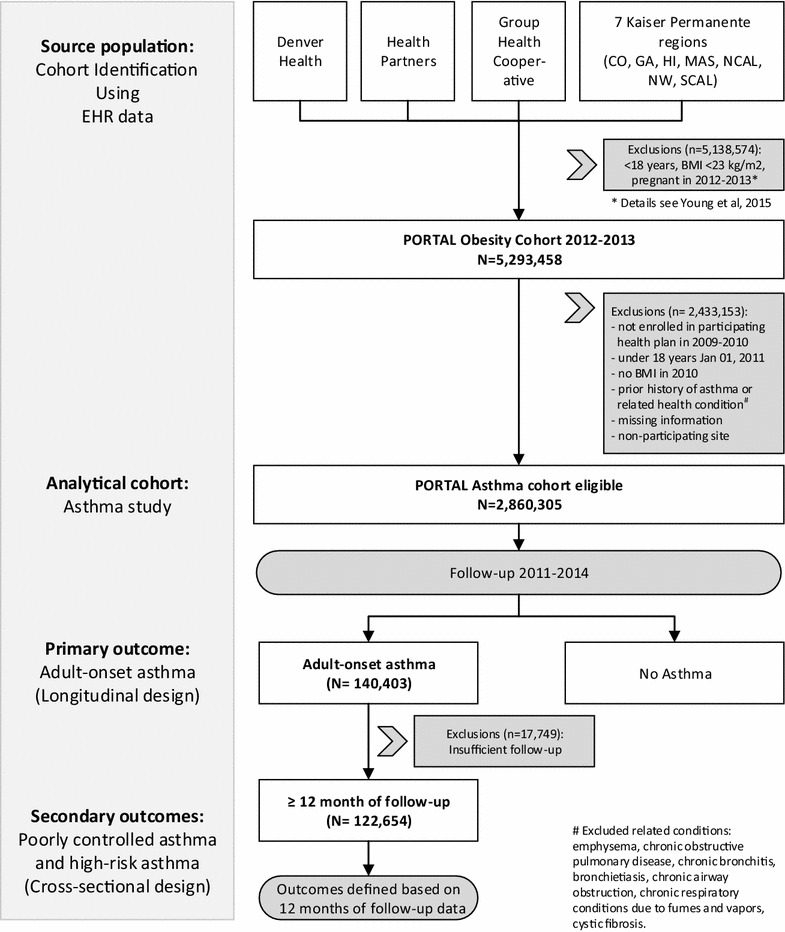
Study flow chart modified from Young et al. [8]. *BMI* body mass index; *EHR* electronic health records; *PORTAL* patients outcomes research to advance learning

The study has a mixed longitudinal (primary study outcome) and cross-sectional (secondary study outcomes) design (Fig. 1). A cohort baseline date was assumed on January 1, 2011. To exclude the possibility of preexisting asthma based on at least 2 years of medical history, we restricted the original PORTAL cohort to individuals who were also enrolled in one of the health plans in 2009 and 2010 (n = 4,568,725). We excluded patients who were not at least 18 years of age at cohort baseline (n = 331,504) or who did not have a measure of weight, height, or BMI in the EHR within a year prior to the cohort baseline (n = 817,674). We then excluded individuals with a history of asthma and other chronic pulmonary diseases (Table 1; n = 559,242). The final analytic cohort consisted of 2,860,305 individuals.

**Table 1:** Exclusion and censoring criteria

Medical condition	International classification of disease, 9th modification ICD-9 diagnosis code
Emphysema	492.x
Chronic obstructive pulmonary disease including chronic bronchitis	490.x–491.x
Bronchiectasis	494.x
Chronic airway obstruction	496.x
Chronic respiratory conditions due to fumes and vapors	506.4
Cystic fibrosis	277.0
Prior history of asthma	493.x

### Weight and height

We used weight and height measurements extracted from outpatient clinic visits before but closest to the index date (at maximum 1 year prior to the index date) to calculate baseline BMI [8]. Weight is routinely measured as part of obtaining vital signs during outpatient clinic visits at PORTAL sites. Height is typically assessed less often, as it is considered to be more static. BMI is calculated within the EHR. A height <4 ft or ≥8 ft or a weight <50 lbs or ≥1000 lbs were considered implausible and removed from the data. Similarly, a calculated BMI <5 kg/m^2^ or ≥90 kg/m^2^ was excluded. A total of 6954 (0.1 %) individuals were excluded from the cohort because they had no biologically plausible weight, height, or BMI values.

We categorized individuals based on their baseline BMI as normal weight (BMI <25 kg/m^2^), overweight (25.0–29.9 kg/m^2^), obese class 1 (30.0–34.9 kg/m^2^), obese class 2 (35.0–39.9 kg/m^2^), obese class 3 (40.0–49.9 kg/m^2^), and obese class 4 (≥50 kg/m^2^) [11, 12]. We classified Asians in the same manner for the main analysis. Because the baseline BMI used for this study was in 2011 not 2012/2013 as for the PORTAL overweight/obesity cohort, the normal weight category includes of individuals with a BMI <23 kg/m^2^ before 2012/2013. For a secondary analysis, we classified Asians as normal weight (BMI <23 kg/m^2^), overweight (23.0–24.9 kg/m^2^), obese class 1 (25.0–29.9 kg/m^2^), obese class 2 (30.0–34.9 kg/m^2^), obese class 3 (35.0–39.9 kg/m^2^), and obese class 4 (≥40 kg/m^2^) [13].

### Race and ethnicity

Based on the self-reported identification from administrative records, individuals were categorized as Asian, Black or African American, Hispanic, Native Hawaiian or other Pacific Islander, American Indian/Alaskan Native (AIAN), non-Hispanic White, or other/unknown [14]. Hispanic ethnicity took priority over any racial category.

### Socioeconomic status

As indicator of socioeconomic status, two measures were used: neighborhood education and insurance through government health care assistance programs.

Neighborhood education was estimated using geospatial entity object codes (geocodes) that linked addresses to 2010 United States census data at the block group level. The probability of high school graduation or less within a block group was used and low neighborhood education defined as the probability of a high school education or less above the population median. Individuals with missing neighborhood education (n = 82,833) were assigned the mean probability of high school graduation for their region.

Information on insurance through government health care assistance programs for individuals with low income and limited resources such as Medicaid (yes/no) was extracted from insurance plan administrative information. Individuals with missing insurance information (n = 853) were assigned to no government health care assistance programs.

### Oral contraceptive use

Because female sex hormones are a risk factor for asthma [3] and are associated with obesity, woman were identified as using oral contraceptives if oral contraceptives were dispensed at least once at any point between 2009 and the end of follow-up for the primary study outcome.

### Definition of study outcomes

The primary study outcome was adult-onset asthma defined as (1) one or more hospitalization or emergency department visit with any diagnosis of asthma [*International Classification of Disease (ICD), 9th modification* ICD-9 diagnosis code 493.x], (2) two or more ambulatory visits with diagnosis of asthma, or (3) at least one visit with a diagnosis of asthma in combination with at least one dispensing of asthma-related medication in the year following the diagnosis [15, 16]. The outcome date was defined as the first date for which a patient met 1 of the above criteria.

The secondary study outcomes were poorly controlled asthma with or without high AMR as indicator for difficulties to achieve asthma control and high-risk asthma. For these secondary study outcomes, the cohort was limited to individuals with adult-onset asthma, at least 12 months of follow-up after the first diagnosis, and medication dispense information available (n = 140,403).

The secondary outcomes were defined based on asthma medications and asthma-related utilization as follows:

Asthma medication dispenses were extracted from the EHR. Medications classified as controllers included inhaled corticosteroids (flunisolide, mometasone, triamcinolone, ciclesonide, fluticasone, budesonide, beclomethasone), combined inhaled corticosteroids/long-acting β-agonists (fluticasone/salmeterol, mometasone/formoterol, budesonide/formoterol), methylxanthines (aminophylline, dyphylline, theophylline), mast cell stabilizer (cromolyn), and leukotriene receptor antagonists (montelukast, zafirlukast, zileuton) [17]. Medications classified as controllers did not include oral corticosteroids. Reliever (rescue) medications included inhaled short-acting β-agonists (albuterol, levalbuterol, metaproterenol) [17]. For persons with asthma and at least 1 year of follow-up, the number and type of medications dispensed during the 1-year period following the incidence date of asthma diagnosis were examined for characterization of asthma severity and control.

An asthma medication ratio was calculated according to the specifications of the National Committee for quality assurance [17]. The asthma medication ratio (AMR) is defined as the number of controller medication fills divided by the sum of the number of controller and rescue medication fills. Both medications were weighted for the number of doses per container to account for differences between products [17, 18]. The AMR generally is calculated over a 1-year period and ranges from 0 to 1 where 1 is ideal (i.e., all controller and no rescue medications). We categorized the AMR as “high” (>=0.5) or “low” (<0.5), which previously has been shown to be associated with patient outcomes [16, 19, 20].

The amount of dispensing of rescue medication over 1 year is an indicator of long-term asthma control [16, 20]. Asthma control was defined based on the number of rescue medication canisters dispensed in the first 12 months after diagnosis and categorized as “poorly controlled” (>=6 canisters) or “adequately controlled” (<6 canisters) [18]. Poorly controlled asthma can be related to factors such as higher asthma severity, inadequate controller prescribing, poor medication adherence, environmental exposures or unaddressed comorbidities [21].

To distinguish between poorly controlled with low or high AMR as indicator for difficulties to achieve asthma control, we divided patients with poorly controlled asthma into those with low (<0.5) and high AMR (>=0.5) [18, 19].

High-risk asthma was defined as having 1 or more asthma-related emergency department visits and/or 1 or more oral steroid dispensing within 7 days of an asthma-related ambulatory visit during the 1-year follow up [18].

### Cohort follow-up

For the primary study outcome, adult-onset asthma, individuals were passively followed through June 30, 2014 using information from the EHR. The follow-up time was calculated from January 1, 2011 until the first occurrence of one of the following events: diagnosis of adult-onset asthma, diagnosis of other chronic pulmonary diseases, the end of health care coverage, death, or the end of follow-up on December 31, 2014.

### Statistical analysis

Demographic and clinical characteristics were summarized as absolute numbers and proportions and reported by weight status. Chi squared tests were used to assess differences in proportions of demographic and clinical characteristics. These characteristics were examined and compared for each site to ensure there were no systematic site-level differences.

The association between baseline weight status and asthma risk was evaluated using hazard ratios (HR) estimated from Cox proportional hazard models with person-time of follow-up as the time scale adjusted for or stratified by age, sex, race/ethnicity, insurance through government health care assistance programs, neighborhood education level, oral contraceptive use, and site. We examined the association of BMI and asthma risk within strata of potential effect modifiers. Tests of linear trends across weight category were conducted by considering weight category as a continuous variable in the multivariate model. To investigate whether the association between asthma risk and BMI category was modified by other potential risk factors for asthma, we performed tests for multiplicative interaction using likelihood-ratio tests. All HRs are displayed with 95 % confidence intervals (CI); reported *P* values for trends and interactions are based on 2-sided tests.

Among adults with adult-onset asthma and at least 12 months of follow-up (Fig. 1), associations among weight status and asthma-specific outcomes were assessed. We also examined how associations were modified by sex, race, and other factors. Due to smaller cell sizes, Asian, Pacific Islander, and AIAN races were combined into one category for these secondary analyses. Odds ratios (OR) for the association between weight status and poorly controlled asthma with low or high AMR, and high-risk asthma were estimated using logistic regression adjusted for age, sex, race/ethnicity, insurance, neighborhood education level, oral contraceptive use and site. Sensitivity analyses were performed excluding persons with missing neighborhood education (n = 82,833) and insurance (n = 853) information as well as removing education and insurance as covariates with essentially unaltered results. All statistical analyses were conducted using SAS version 9.3 (SAS Institute, Inc., Cary, NC, USA).

## Results

The PORTAL cohort is ethnically and racially diverse with 49.4 % non-Hispanic Whites, 23.0 % Hispanics, 11.4 % Blacks and 10.5 % Asians (Table 2). With increasing weight class, individuals were more likely to be in the age group between 40–60 years of age, female, non-White, a recipient of care through a government insurance program for individuals with low income and limited resources such as Medicaid, and a resident of a neighborhood with higher educational attainment.

**Table 2:** Distribution of baseline demographic characteristics of PORTAL patients (n = 2,860,305)

	Total population	BMI (kg/m^2^)					
		<25	25.0–29.9	30.0–34.9	35.0–39.9	40.0–49.9	≥50
N (%)							
Age (years)							
18–39	700,548 (24.5 %)	187,257 (31.2 %)	260,014 (22.5 %)	141,290 (21.5 %)	64,996 (23.7 %)	39,778 (26.6 %)	7213 (30.1 %)
40–65	1,507,052 (52.7 %)	269,106 (44.9 %)	605,983 (52.4 %)	369,320 (56.3 %)	159,459 (58.1 %)	88,592 (59.3 %)	14,592 (60.9 %)
65+	652,705 (22.8 %)	143,604 (23.9 %)	290,840 (25.1 %)	145,162 (22.1 %)	50,046 (18.2 %)	20,906 (14.0 %)	2147 (9.0 %)
Sex							
Male	1,307,645 (45.7 %)	223,739 (37.3 %)	587,681 (50.8 %)	323,679 (49.4 %)	114,094 (41.6 %)	50,906 (34.1 %)	7546 (31.5 %)
Female	1,552,660 (54.3 %)	376,228 (62.7 %)	569,156 (49.2 %)	332,093 (50.6 %)	160,407 (58.4 %)	98,370 (65.9 %)	16,406 (68.5 %)
Race/ethnicity							
Non-Hispanic White	1,413,998 (49.4 %)	305,329 (50.9 %)	575,541 (49.8 %)	318,138 (48.5 %)	132,652 (48.3 %)	71,183 (47.7 %)	11,155 (46.6 %)
Hispanic	656,642 (23.0 %)	105,589 (17.6 %)	263,032 (22.7 %)	173,604 (26.5 %)	72,069 (26.3 %)	36,972 (24.8 %)	5376 (22.4 %)
Black	327,148 (11.4 %)	46,157 (7.7 %)	113,792 (9.8 %)	87,652 (13.4 %)	44,754 (16.3 %)	29,181 (19.5 %)	5612 (23.4 %)
Asian	301,722 (10.5 %)	108,670 (18.1 %)	138,957 (12.0 %)	40,504 (6.2 %)	9845 (3.6 %)	3422 (2.3 %)	324 (1.4 %)
Pacific Islander	37,719 (1.3 %)	7335 (1.2 %)	13,976 (1.2 %)	8660 (1.3 %)	4321 (1.6 %)	2821 (1.9 %)	606 (2.5 %)
AIAN	14,552 (0.5 %)	2493 (0.4 %)	5189 (0.4 %)	3675 (0.6 %)	1850 (0.7 %)	1115 (0.7 %)	230 (1.0 %)
Other/unknown	108,524 (3.8 %)	24,394 (4.0 %)	46,350 (4.0 %)	23,539 (3.6 %)	9010 (3.3 %)	4582 (3.1 %)	649 (2.7 %)
Health care assistance							
No	2,809,762 (98.2 %)	590,175 (98.4 %)	1,139,813 (98.5 %)	643,938 (98.2 %)	268,196 (97.7 %)	144,760 (97.0 %)	22,880 (95.5 %)
Yes	50,543 (1.8 %)	9792 (1.6 %)	17,024 (1.5 %)	11,834 (1.8 %)	6305 (2.3 %)	4516 (3.0 %)	1072 (4.5 %)
Neighborhood education							
Low	1,387,326 (48.5 %)	328,707 (54.8 %)	582,814 (50.4 %)	295,934 (45.1 %)	114,239 (41.6 %)	57,322 (38.4 %)	8310 (34.7 %)
High	1,472,979 (51.5 %)	271,260 (45.2 %)	574,023 (49.6 %)	359,838 (54.9 %)	160,262 (58.4 %)	91,954 (61.6 %)	15,642 (65.3 %)
Oral contraceptives (women only)							
Yes	467,551 (16.3 %)	133,053 (22.2 %)	168,619 (14.6 %)	91,033 (13.9 %)	43,140 (15.7 %)	26,925 (18.0 %)	4781 (20.0 %)
No	2,392,754 (83.7 %)	466,914 (77.8 %)	988,218 (85.4 %)	564,739 (86.1 %)	231,361 (84.3 %)	122,351 (82.0 %)	19,171 (80.0 %)

Chi squared tests were used to assess differences in proportions of demographic and clinical characteristics across different weight classes. *P* < 0.001 for all demographic and clinical characteristics shown

*AIAN* American Indian/American Native; *BMI* body mass index

**Fig. 2:**
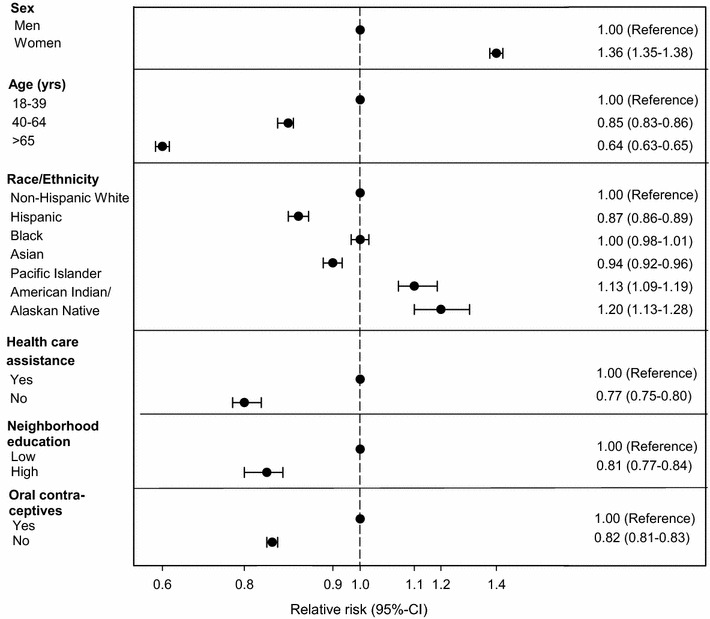
Risk for asthma by age, sex, race and sociodemographic characteristics

During 11,753,217 person-years of follow-up (median follow-up time 4.5 years), we identified 140,403 individuals who met the study definition of adult-onset asthma. In multivariate-adjusted models, a new diagnosis of asthma was more common among women, younger individuals, members of selected racial groups, and persons with lower socioeconomic status (SES) (Fig. 2).

The risk for adult-onset asthma was strongly and positively associated with BMI categories. In multivariate-adjusted models, the hazard of developing adult-onset asthma progressively increased with higher BMI categories, from a 12 % increase among persons with a BMI of 25.0–29.9 kg/m^2^ (HR 1.12, 95 % CI 1.10, 1.14) to an almost 250 % increase among persons with a BMI ≥50 kg/m^2^ (HR 2.49, 95 % CI 2.38, 2.60) (Table 3).

**Table 3:** Adjusted HR of asthma according to weight class by race, age, and sex

		BMI (kg/m^2^)											*P* for trend	*P* for interaction
	No. of cases	<25	25.0–29.9		30.0–34.9		35.0–39.9		40.0–49.9		≥50			
		Ref	HR	95 % CI	HR	95 % CI	HR	95 % CI	HR	95 % CI	HR	95 % CI		
All ages	140,403	1.00	1.12	1.10, 1.14	1.37	1.35, 1.40	1.64	1.61, 1.68	1.97	1.92, 2.01	2.49	2.38, 2.60	<0.0001	–
Age (years)														
18–39	41,812	1.00	1.03	1.00, 1.06	1.18	1.15, 1.22	1.34	1.30, 1.39	1.57	1.51, 1.64	1.89	1.75, 2.04	<0.0001	<0.001
40–65	74,755	1.00	1.18	1.15, 1.20	1.46	1.42, 1.49	1.77	1.72, 1.82	2.17	2.11, 2.24	2.79	2.64, 2.94	<0.0001	
65+	23,836	1.00	1.25	1.21, 1.30	1.65	1.59, 1.72	2.11	2.01, 2.21	2.47	2.32, 2.63	3.52	3.05, 4.06	<0.0001	
Sex														
Male	51,018	1.00	1.07	1.04, 1.10	1.26	1.23, 1.30	1.50	1.45, 1.56	1.78	1.70, 1.85	2.19	2.01, 2.39	<0.0001	<0.001
Female	89,385	1.00	1.14	1.12, 1.16	1.43	1.40, 1.46	1.71	1.67, 1.75	2.05	2.00, 2.10	2.59	2.47, 2.72	<0.0001	
Race/ethnicity														
Non-Hispanic White	73,049	1.00	1.13	1.10, 1.15	1.38	1.35, 1.41	1.65	1.60, 1.69	1.99	1.93, 2.05	2.55	2.40, 2.70	<0.0001	<0.001
Hispanic	28,386	1.00	1.07	1.03, 1.11	1.29	1.24, 1.34	1.59	1.53, 1.67	1.90	1.81, 2.00	2.29	2.08, 2.53	<0.0001	
Black	16,855	1.00	1.01	0.96, 1.06	1.22	1.16, 1.28	1.41	1.33, 1.49	1.69	1.59, 1.79	2.04	1.85, 2.24	<0.0001	
Asian	14,623	1.00	1.23	1.19, 1.28	1.62	1.54, 1.70	2.08	1.93, 2.25	2.34	2.09, 2.62	4.45	3.39, 5.83	<0.0001	
Pacific Islander	2332	1.00	1.00	0.88, 1.13	1.21	1.07, 1.38	1.39	1.20, 1.61	1.44	1.22, 1.69	2.07	1.60, 2.68	<0.0001	
American Indian/American native	994	1.00	1.13	0.92, 1.38	1.32	1.07, 1.63	1.58	1.25, 1.99	2.14	1.68, 2.72	2.61	1.78, 3.82	<0.0001	
Other/unknown	4164	1.00	1.08	1.00, 1.18	1.32	1.20, 1.45	1.56	1.39, 1.75	1.95	1.71, 2.23	2.42	1.82, 3.21	<0.0001	
Men only														
Age (years)														
18–39	15,196	1.00	0.98	0.94, 1.02	1.10	1.05, 1.15	1.20	1.13, 1.28	1.39	1.29, 1.50	1.66	1.43, 1.93	<0.0001	<0.001
40–65	26,659	1.00	1.13	1.08, 1.17	1.35	1.29, 1.41	1.63	1.55, 1.72	1.96	1.84, 2.08	2.48	2.22, 2.78	<0.0001	
65+	9163	1.00	1.20	1.13, 1.28	1.50	1.41, 1.61	1.96	1.80, 2.13	2.46	2.19, 2.77	3.29	2.38, 4.55	<0.0001	
Race/ethnicity														
Non-Hispanic White	27,157	1.00	1.09	1.05, 1.13	1.28	1.23, 1.33	1.54	1.46, 1.61	1.85	1.75, 1.96	2.17	1.92, 2.45	<0.0001	<0.001
Hispanic	9889	1.00	0.97	0.90, 1.03	1.13	1.06, 1.21	1.39	1.28, 1.50	1.61	1.46, 1.77	1.84	1.51, 2.23	<0.0001	
Black	5091	1.00	0.95	0.87, 1.03	1.11	1.01, 1.21	1.16	1.04, 1.29	1.39	1.23, 1.57	1.68	1.35, 2.09	<0.0001	
Asian	5731	1.00	1.20	1.13, 1.28	1.56	1.44, 1.69	2.14	1.89, 2.42	2.18	1.79, 2.66	4.41	2.81, 6.95	<0.0001	
Pacific Islander	916	1.00	0.99	0.80, 1.22	1.12	0.90, 1.40	1.20	0.93, 1.55	1.25	0.94, 1.66	2.08	1.41, 3.08	<0.0001	
AIAN	304	1.00	0.83	0.58, 1.18	1.00	0.69, 1.45	1.30	0.85, 1.97	1.62	1.00, 2.61	2.55	1.19, 5.45	<0.0001	
Other/unknown	1930	1.00	1.04	0.92, 1.18	1.19	1.03, 1.37	1.38	1.15, 1.66	1.74	1.39, 2.18	3.55	2.36, 5.34	<0.0001	
Women only														
Age (years)														
18–39	26,616	1.00	1.05	1.02, 1.09	1.23	1.19, 1.27	1.42	1.36, 1.48	1.66	1.58, 1.74	1.99	1.83, 2.18	<0.0001	<0.001
40–65	48,096	1.00	1.19	1.16, 1.22	1.51	1.47, 1.55	1.82	1.77, 1.89	2.25	2.17, 2.33	2.88	2.71, 3.07	<0.0001	
65+	14,673	1.00	1.27	1.22, 1.34	1.74	1.66, 1.83	2.18	2.05, 2.32	2.48	2.30, 2.67	3.60	3.06, 4.22	<0.0001	
Race/ethnicity														
Non-Hispanic White	45,892	1.00	1.14	1.11, 1.17	1.44	1.40, 1.48	1.70	1.65, 1.76	2.05	1.97, 2.12	2.68	2.51, 2.87	<0.0001	<0.001
Hispanic	18,497	1.00	1.11	1.06, 1.16	1.36	1.30, 1.42	1.68	1.59, 1.77	2.01	1.89, 2.13	2.46	2.20, 2.76	<0.0001	
Black	11,764	1.00	1.04	0.98, 1.11	1.29	1.21, 1.37	1.53	1.43, 1.64	1.82	1.69, 1.95	2.18	1.96, 2.43	<0.0001	
Asian	8892	1.00	1.26	1.20, 1.32	1.65	1.55, 1.76	2.05	1.86, 2.25	2.42	2.10, 2.78	4.44	3.17, 6.23	<0.0001	
Pacific Islander	1416	1.00	0.99	0.85, 1.16	1.26	1.07, 1.49	1.51	1.25, 1.81	1.55	1.26, 1.90	2.04	1.44, 2.88	<0.0001	
AIAN	690	1.00	1.29	1.01, 1.64	1.48	1.15, 1.91	1.71	1.29, 2.26	2.37	1.78, 3.14	2.73	1.75, 4.26	<0.0001	
Other/unknown	2234	1.00	1.10	0.99, 1.24	1.43	1.26, 1.62	1.69	1.46, 1.97	2.09	1.77, 2.47	1.86	1.26, 2.77	<0.0001	
Oral contraceptives (women only)														
Yes	32,399	1.00	1.09	1.06, 1.12	1.34	1.30, 1.38	1.54	1.48,1.60	1.80	1.72,1.88	2.06	1.89, 2.24	<0.0001	<0.001
No	56,986	1.00	1.19	1.16, 1.21	1.51	1.47, 1.55	1.84	1.78,1.89	2.23	2.16, 2.30	2.97	2.80, 3.15	<0.0001	

Hazard ratios were estimated from Cox proportional hazard models adjusted for age (continuous), sex (male, female), race/ethnicity (non-Hispanic White, Hispanic, Black, Asian, Pacific Islander, AIAN, other or unknown), insurance through government health care assistance programs (yes/no), neighborhood education level (continuous probability of education below high school), oral contraceptive use (yes/no), and site (Denver Health, Group Health Cooperative, HealthPartners and seven Kaiser Permanente regions). Tests of linear trend across weight category were conducted by considering weight category as a continuous variable in the multivariate model

*CI* confidence intervals; *HR* hazard ratios; *AIAN* American Indian/Alaskan Native

To evaluate whether the association between BMI and adult-onset asthma was modified by age, sex and race, we tested for interaction effects and repeated our analyses within subgroups defined by those variables (Table 3). The association of BMI to asthma incidence appeared to be stronger among women than among men, and in older (40–65 and 65+ year) compared with younger individuals (18–39 year; *P* value for interaction <0.001).

**Fig. 3:**
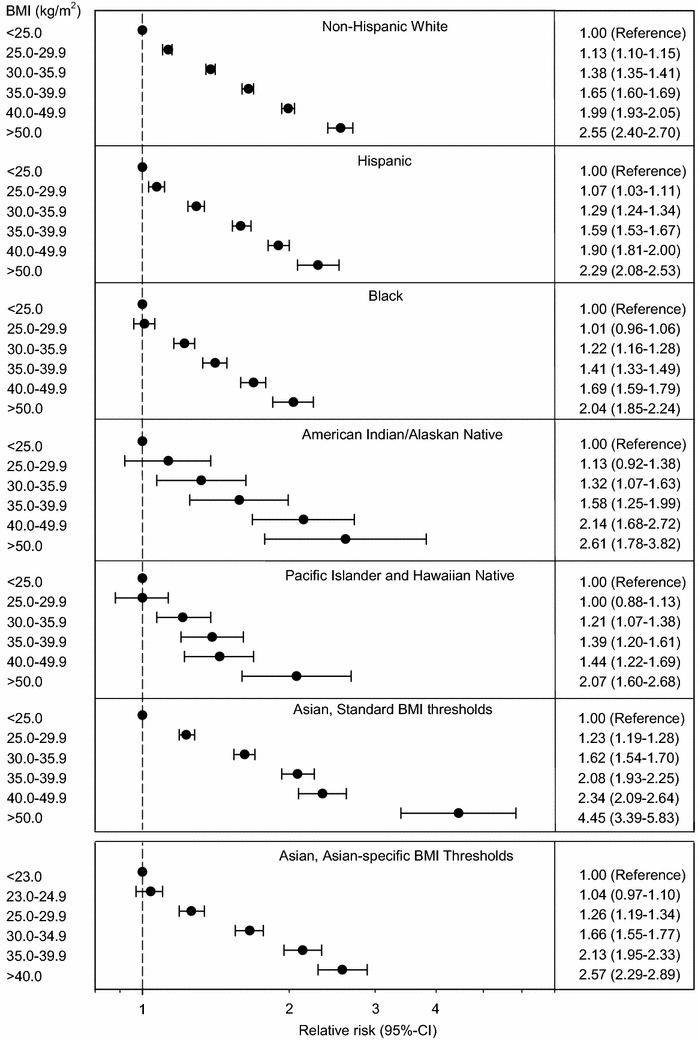
Risk for asthma across the different weight class

The association of BMI and asthma incidence also appeared to be stronger among Asians and weaker among Blacks than among other races/ethnicities (*P* value for interaction < 0.001, Fig. 3). When applying Asian-specific BMI thresholds to compare the risk for asthma across the different weight classes, the association of obesity to asthma was comparable between Asians and non-Hispanic Whites (Fig. 3).

For women, the association of BMI to adult-onset asthma was stronger among oral contraceptives non-users than among users (*P* value for interaction < 0.001). For example, the HR was 2.97 (95 % CI 2.80, 3.15) for non-users and 2.06 (95 % CI 1.89, 2.24) for users with a BMI ≥50 kg/m^2^ when compared with their normal weight counterparts (Table 3).

The association of body weight to adult-onset asthma in all subgroups defined by age and race was roughly comparable among men and women after further stratifying by sex (Table 3).

In individuals with adult-onset asthma and at least 1 year of follow up, we examined the association of body weight class and asthma outcomes. Poorly controlled asthma (3083 cases) including poorly controlled asthma with high AMR (758 cases), and high-risk asthma (17,204 cases) were more prevalent at higher BMI categories (Table 4). The OR for poorly controlled asthma for individuals with a BMI <25 kg/m^2^, 25.0–29.9 kg/m^2^, 30.0–34.9 kg/m^2^, 40.0–49.9 kg/m^2^, and ≥50 kg/m^2^ were 1.00, 0.97, 1.03, 1.13, 1.13 and 1.46 (95 % CI 1.13, 1.88), respectively. The OR for poorly controlled asthma with high AMR for individuals with a BMI <25 kg/m^2^, 25.0–29.9 kg/m^2^, 30.0–34.9 kg/m^2^, 40.0–49.9 kg/m^2^, and ≥50 kg/m^2^ were 1.00, 1.12, 1.21, 1.26, 1.25 and 2.08 (95 % CI 1.31, 3.31), respectively. The OR for high-risk asthma were 1.00, 1.03, 1.17, 1.30, 1.37, and 1.63 (95 % CI 1.45, 1.84), respectively.

**Table 4:** Adjusted odds ratio of poorly controlled asthma with or without high AMR and high-risk asthma

		BMI (kg/m^2^)											*P* for trend	*P* for interaction
	No. of cases	<25	25.0–29.9		30.0–34.9		35.0–39.9		40.0–49.9		≥50			
		Ref	OR	95 % CI	OR	95 % CI	OR	95 % CI	OR	95 % CI	OR	95 % CI		
Poorly controlled asthma (any AMR)														
All ages	3083	1.00	0.97	0.87, 1.08	1.03	0.91, 1.15	1.13	0.99, 1.29	1.13	0.97, 1.31	1.46	1.13, 1.88	0.001	–
Age (years)														
18–39	721	1.00	1.17	0.96, 1.43	1.15	0.92, 1.44	1.30	0.99, 1.70	1.17	0.85, 1.59	0.85	0.43, 1.68	0.268	<0.001
40–65	1842	1.00	0.91	0.78, 1.05	1.00	0.86, 1.17	1.04	0.87, 1.24	1.13	0.93, 1.37	1.42	1.04, 1.94	0.005	
65+	520	1.00	0.85	0.65, 1.10	0.87	0.66, 1.15	1.17	0.85, 1.61	0.87	0.57, 1.35	3.24	1.75, 6.01	0.076	
Sex														
Male	1367	1.00	1.03	0.87, 1.22	1.12	0.93, 1.34	1.27	1.03, 1.58	1.26	0.98, 1.63	1.48	0.92, 2.38	0.002	0.695
Female	1716	1.00	0.94	0.82, 1.08	0.97	0.84, 1.13	1.06	0.89, 1.26	1.07	0.88, 1.28	1.44	1.07, 1.95	0.039	
Race/ethnicity														
Non-Hispanic White	1845	1.00	1.02	0.89, 1.17	0.94	0.81, 1.09	1.05	0.89, 1.25	1.10	0.90, 1.33	1.27	0.90, 1.79	0.264	0.055
Hispanic	459	1.00	0.86	0.63, 1.16	1.05	0.77, 1.44	1.16	0.82, 1.64	1.18	0.80, 1.76	1.73	0.90, 3.33	0.017	
Black	375	1.00	0.64	0.44, 0.92	0.94	0.66, 1.33	1.00	0.69, 1.47	0.92	0.61, 1.38	1.38	0.77, 2.48	0.057	
Asian/PI/AIAN^a^	293	1.00	1.05	0.77, 1.42	1.45	1.03, 2.04	1.41	0.89, 2.25	1.02	0.50, 2.06	2.86	1.20, 6.82	0.018	
Other/unknown	111	1.00	1.14	0.65, 1.98	1.23	0.67, 2.27	1.52	0.75, 3.07	1.31	0.56, 3.07	–	–	0.477	
Poorly controlled asthma with high AMR^b^														
All ages	758	1.00	1.12	0.90, 1.41	1.21	0.95, 1.53	1.26	0.95, 1.65	1.25	0.92, 1.71	2.08	1.31, 3.31	0.009	–
Age (years)														
18–39	124	1.00	1.39	0.84, 2.28	1.65	0.97, 2.81	1.55	0.81, 2.99	0.84	0.34, 2.08	1.24	0.29, 5.31	0.572	0.075
40–65	508	1.00	1.14	0.85, 1.52	1.21	0.89, 1.64	1.18	0.83, 1.68	1.48	1.03, 2.14	2.32	1.36, 3.95	0.005	
65+	126	1.00	0.86	0.52, 1.44	0.89	0.51, 1.56	1.28	0.69, 2.39	0.52	0.18, 1.53	2.03	0.47, 8.82	0.812	
Sex														
Male	314	1.00	1.33	0.92, 1.94	1.23	0.82, 1.85	1.53	0.96, 2.43	1.52	0.88, 2.64	2.48	1.03, 5.97	0.054	0.502
Female	444	1.00	1.00	0.75, 1.33	1.22	0.91, 1.64	1.12	0.79, 1.58	1.13	0.77, 1.64	1.87	1.08, 3.24	0.076	
Race/ethnicity														
Non-Hispanic White	461	1.00	1.31	0.98, 1.75	1.17	0.86, 1.60	1.12	0.78, 1.62	1.39	0.94, 2.04	2.15	1.19, 3.87	0.141	0.357
Hispanic	106	1.00	0.81	0.42, 1.57	1.19	0.62, 2.28	1.20	0.58, 2.49	1.27	0.57, 2.87	1.33	0.30, 5.96	0.171	
Black	82	1.00	0.75	0.33, 1.68	1.06	0.49, 2.31	1.24	0.55, 2.81	0.78	0.31, 2.00	1.20	0.32, 4.53	0.615	
Asian/PI/AIAN^a^	80	1.00	0.92	0.52, 1.62	1.26	0.66, 2.42	1.68	0.73, 3.84	0.46	0.06, 3.48	6.22	1.76, 21.94	0.098	
Other/unknown	29	1.00	1.14	0.35, 3.71	1.96	0.59, 6.47	2.48	0.65, 9.51	0.85	0.09, 7.74	–	–	0.395	
High-risk asthma														
All ages	17,204	1.00	1.03	0.98, 1.08	1.17	1.11, 1.24	1.30	1.23, 1.38	1.37	1.29, 1.47	1.63	1.45, 1.84	<0.0001	–
Age (years)														
18–39	4998	1.00	0.93	0.86, 1.01	1.04	0.95, 1.14	1.13	1.01, 1.26	1.35	1.20, 1.51	1.60	1.30, 1.97	<0.0001	<0.001
40–65	8986	1.00	1.11	1.03, 1.20	1.30	1.21, 1.41	1.48	1.35, 1.61	1.50	1.37, 1.65	1.68	1.44, 1.97	<0.0001	
65+	3220	1.00	1.06	0.95, 1.19	1.16	1.02, 1.31	1.25	1.08, 1.45	1.17	0.97, 1.40	1.84	1.25, 2.70	<0.0001	
Sex														
Male	5681	1.00	0.94	0.86, 1.02	1.10	1.01, 1.21	1.24	1.11, 1.38	1.28	1.12, 1.46	1.69	1.32, 2.16	<0.0001	<0.001
Female	11,523	1.00	1.08	1.01, 1.14	1.20	1.13, 1.28	1.33	1.24, 1.43	1.41	1.31, 1.53	1.64	1.43, 1.87	<0.0001	
Race/ethnicity														
Non-Hispanic White	8776	1.00	1.04	0.97, 1.11	1.18	1.10, 1.27	1.34	1.23, 1.45	1.37	1.25, 1.51	1.69	1.43, 2.00	<0.0001	<0.001
Hispanic	3666	1.00	0.90	0.81, 1.01	1.03	0.92, 1.16	1.18	1.04, 1.35	1.21	1.04, 1.40	1.20	0.90, 1.59	<0.0001	
Black	2412	1.00	0.88	0.76, 1.03	1.08	0.93, 1.26	1.04	0.88, 1.23	1.20	1.00, 1.42	1.48	1.14, 1.92	<0.0001	
Asian/PI/AIAN^a^	2030	1.00	1.31	1.16, 1.48	1.45	1.25, 1.67	1.56	1.29, 1.89	1.91	1.50, 2.42	2.32	1.52, 3.56	<0.0001	
Other/unknown	320	1.00	0.74	0.54, 1.02	0.79	0.55, 1.13	1.27	0.85, 1.89	1.27	0.80, 2.01	1.26	0.48, 3.31	0.063	

ORs were estimated from logistic regression models adjusted for age (continuous), sex (male, female), race/ethnicity (non-Hispanic White, Hispanic, Black, Asian/PI/AIAN, or other/unknown), insurance through government health care assistance programs (yes/no), neighborhood education level (continuous probability of education below high school), oral contraceptive use (yes/no), and site (Denver Health, Group Health Cooperative, HealthPartners and seven Kaiser Permanente regions). Tests of linear trend across weight category were conducted by considering weight category as a continuous variable in the multivariate model

*AIAN* American Indian/American Native; *CI* confidence interval; *OR* odds ratio; *PI* Pacific Islander

^a^Due to sample size limitations, these groups were combined for the outcomes related to asthma control

^b^Subgroup of poorly controlled asthma

The magnitude of the association between BMI and poorly controlled asthma (*P* value for interaction < 0.001) and high-risk asthma (*P* value for interaction < 0.001) was stronger among younger compared with older individuals, while the magnitude of the association between BMI and poorly controlled asthma with high AMR was comparable across all age groups (*P* value for interaction = 0.075). The magnitude of the association between BMI and high-risk asthma (*P* value for interaction < 0.001) was stronger among Asians, Pacific Islanders and AIAN than among other races, while the magnitude of the association between BMI and poorly controlled asthma (*P* value for interaction = 0.055), especially for poorly controlled asthma with high AMR (*P* value for interaction = 0.357) was comparable across all racial/ethnic groups.

## Discussion

Obesity has been previously recognized as a risk factor for adult-onset asthma. We demonstrated that excessive body weight was associated with a 40 % higher risk of asthma for individuals affected by obesity with a BMI of 30.0–34.9 kg/m^2^ and almost 250 % higher risk for individuals with a BMI of over 50 kg/m^2^ compared to individuals with a normal weight BMI <25 kg/m^2^. We also found that the magnitude of this association may be altered by age, sex, and race. Women, older adults, and individuals of Asian origin carried a higher obesity-related risk to develop asthma during adulthood than other population groups. Furthermore, among individuals with asthma, greater BMI was associated with higher odds for poorly controlled and high-risk asthma across all racial and ethnic groups. In addition, the obesity-related risk for poorly controlled and high-risk asthma increased with age, and was marginally to significantly higher among individuals of Asian origin.

Asthma is a heterogeneous disease with many phenotypes, and age at disease onset is an important factor in separating the phenotypes. Patients with childhood-onset asthma are typically atopic with a relatively good prognosis [22]. In contrast, patients with adult-onset asthma are most often non-atopic females without a family history of asthma or allergies, have a less favorable prognosis, and are more likely to develop persistent airflow limitation [3, 23, 24]. Asthma in patients affected by obesity, especially in women, has been identified as a distinct phenotype characterized by frequent symptoms, frequent exacerbations and high health care utilization [24]. The results from our study contribute to the evidence that asthma is not only more likely to develop in individuals affected by obesity, but also that existing asthma is complicated by obesity, with some demographic subgroups experiencing worse asthma control than others. With more than one-third of U. S. adults considered obese, clinical care approaches, asthma risk stratification algorithms and population management strategies may benefit from taking into account that obesity can alter asthma outcomes for some population groups.

Several cross-sectional and prospective studies, as well as systematic reviews and meta-analyses, provide compelling evidence that obesity increases the risk of asthma in children and adults [4, 25–27]. However, only a few studies were able to distinguish between child- and adult-onset of asthma and provide estimates for the magnitude of the association of obesity class 2 and higher [4]. According to a meta-analysis based on prospective studies, the odds of developing asthma for individuals with obesity compared to those with normal weight were twice as high (OR 1.92, 95 % CI 1.43–2.59) [4]. Cross-sectional data from the National Health and Nutrition Examination Survey (NHANES), 2001–2004, showed an OR of 1.80 (95 % CI 1.40–2.30) for a BMI of 30.0–34.9 kg/m^2^, and 2.40 (95 % CI 1.60–3.70) for a BMI >40.0 kg/m^2^ compared to their normal weight counterparts [28]. In the Black Women’s Health Study, the incidence rate ratios ranged from 1.26 (95 % CI 1.05–1.51) for women with overweight to 2.85 (95 % CI 2.19–3.72) for women with a BMI ≥40 kg/m^2^ when compared to women with a BMI of 20–24.9 kg/m^2^ [29]. Compared to other studies [4, 28, 29], our HRs are slightly lower, but this can be partially explained by the higher BMI of our normal weight control group (23.0–24.9 as opposed to 19.0–24.9).

Several mechanisms for the contribution of obesity to asthma risk have been implicated [3, 30]. Obesity is associated with increased levels of adipokines, which may affect the airway directly, rather than through increased airway inflammation [31]. Additionally, mechanical factors such as reduced functional residual capacity that can occur with more severe obesity may result in expiratory flow limitation and airway closure [32].

Sex has been previously shown to alter the association between obesity and asthma. In a meta-analyses, the odds for asthma among women with overweight and obesity were almost 70 % higher (OR 1.68, 95 % CI 1.45, 1.94) than that found for men with overweight and obesity (1.46, 95 % CI 1.05, 2.02) compared to their counterparts with normal weight [4]. Extending these findings, we observed an asthma risk increase between 43 and 260 % for women and between 26 and 220 % for men with obesity class 1 to class 4 compared to their counterparts with normal weight. While both sexes in our study showed a strong risk increase with higher BMI, the association was significantly altered by sex, showing a higher obesity-related burden for asthma in women. The obesity-related risk for asthma was also modified by age, showing an increased risk with higher age in both, men and women. A potential role of hormonal factors as explanation of sex differences is supported by the effect modification of oral contraceptive use among women. The obesity-related risk for asthma was more pronounced in women who did not use oral contraceptives (OR 2.97, 95 % CI 2.80, 3.15) than in those taking oral contraceptives (OR 2.06, 95 % CI 1.89, 2.24). Results from other studies indicated that oral contraceptive use lowered the risk for asthma in girls during puberty [33] and decreased the risk for asthma and asthma exacerbations in Scottish women [34].

One previous study investigated the effect modification of race/ethnicity on the association between BMI and risk of asthma [35]. Results from that study suggested that obesity is a strong risk factor for asthma among Blacks (OR for BMI ≥30 kg/m^2^ = 2.90, 95 % CI 1.20, 7.00) and Hispanic men (OR for BMI ≥30 kg/m^2^ = 2.70, 95 % CI 1.10, 6.30) but not in non-Hispanic White men (OR for BMI ≥30 kg/m^2^ = 1.00, 95 % CI 0.60, 1.70) compared to their counterparts with normal weight of the same race/ethnicity. However, the statistical power was limited due to a relatively small sample size for minority men [35]. These findings are not consistent with our results. We noted a comparable obesity–related burden across most races/ethnicities in men and women except for individuals of Asian origin and Blacks. While Asians appeared to have a slightly lower overall risk for asthma compared to Non-Hispanic Whites in the present study, they exhibited a greater increase in asthma risk with increasing BMI than other races/ethnicities. However, when applying Asian-specific BMI thresholds to define overweight and obesity, the relation of obesity to asthma risk was comparable with most other races. In Blacks, the increase in asthma risk associated with higher BMI appeared to be smaller than in other races.

While asthma is a common comorbidity among persons with obesity, our study suggests that obesity not only increases the risk for asthma, but also adversely affects asthma severity and control. Increasing obesity was associated with less favorable asthma outcomes across all racial and ethnic groups. But similar to the risk of developing adult-onset asthma, the risk associated with obesity class 4 for poorly controlled and high risk asthma was greater in adults who were elderly, and was marginally to significantly greater among individuals of Asian origin when using standard BMI thresholds. In contrast, increasing obesity was associated with poorly controlled asthma with high AMR across all subgroups.

Several biological and non-biological mechanisms have been suggested to explain a relationship between obesity and poor asthma outcomes, including reduced corticosteroid responsiveness in obese individuals [36–38], the influence of immunomodulatory adipokines [39, 40], deleterious effects on pulmonary mechanics [39], low vitamin D levels [41, 42], and obesity-related comorbidities such as gastro-esophageal reflux disease (GERD) and depression [40, 43]. However, a recent study suggested that obesity was associated with poor asthma control even after adjusting for GERD, depression, and corticosteroid use [15]. No studies could be identified that investigated the modification of association between obesity and asthma outcomes by race and ethnicity, age, and sex.

We noted several potential limitations. Due to the inclusion criteria for the PORTAL obesity cohort, individuals were required to have a BMI of at least 23.0 kg/m^2^ in 2012 or 2013. Thus, our study population may not reflect normal weight individuals ranging from 19.0–22.9 kg/m^2^ who are included in most other studies. However, the difference in the range of normal weight will lead to an underestimation of the risk associated with high BMI. Furthermore, this is unlikely to affect the interactions that were the focus of the present study. A unique strength of our study is that we examined the risk for asthma among individuals with severe obesity and examined relevant subgroups stratified by sex, age, and race. Although we were not able to account for changes in the BMI during follow-up, this is unlikely to influence the results, considering the relatively short follow-up and the time of exposure to obesity.

Our definition of adult-onset may have resulted in misclassification of recrudescent pediatric-onset asthma as adult-onset asthma, especially in younger adults. However, the misclassification is unlikely to be differential across weight classes and, therefore, should not affect the nature or the magnitude of the results presented here.

Several studies have suggested that asthma, especially adult-onset asthma, may be overdiagnosed in individuals affected by obesity [44, 45]. Individuals with asthma and obesity have a lower lung function and more comorbidities compared to individuals with asthma who are normal weight; if making urgent visits for respiratory symptoms, individuals affected by obesity are more likely to receive a misdiagnosis of asthma [46]. For the present study, a potential overdiagnosis of asthma in individuals affected by obesity when seeking urgent care would result in an overestimation of the association between obesity and asthma, especially the association between obesity and high-risk asthma. We can also not exclude that differential utilization of health care service such as higher utilization among women or lower utilization among Hispanics or Blacks may have contributed to differential chance of diagnosis with asthma.

Moreover, the relatively short follow-up times may mask some effect modification that may be apparent over longer periods of time. Generally, a short follow-up is likely to bias results towards the null. Future studies with a longer follow-up period are required to identify those effect modifications.

A small proportion of the study population was classified as other or unknown race/ethnicity. Incomplete assessment of race/ethnicity is usually caused by shorter enrollment [14, 47] and unlikely to be differential across different weight classes and, therefore, unlikely to affect the results. In addition, risk estimates of individuals classified as other or unknown were roughly comparable to the largest represented racial groups, i.e., non-Hispanic Whites, Hispanic, and Blacks, and unlikely to mask a significant effect modification in a small racial subgroup with differential classification as other or unknown racial origin.

## Conclusions

We found that the associations between obesity and asthma and asthma control were significantly altered by age, sex, and race. Among individuals with asthma, increasing obesity was associated with poorer asthma outcomes, which was more pronounced in adults who were older. Understanding the role of obesity in the development of adult-onset asthma will help to improve asthma treatment algorithms, design patient-centered outcome studies to better understand patient barriers to asthma control, and to develop targeted interventions.

## Abbreviations

AIAN: American Indian/Alaskan Native
AMR: asthma medication ratio
BMI: body mass index
CI: confidence intervals
EHR: electronic health record
GERD: gastro-esophageal reflux disease
HR: hazard ratios
ICD: International Classification of Disease
IRB: Institutional Review Board
KPSC: Kaiser Permanente Southern California
NHANES: National Health and Nutrition Examination Survey
PORTAL: patients outcomes research to advance learning
OR: odds ratio
SES: socioeconomic status
WHO: World Health Organization
